# The REFRACT trial: implementation of Bayesian power priors in a randomised, sequential phase II adaptive platform trial

**DOI:** 10.1186/s12874-025-02575-5

**Published:** 2025-05-03

**Authors:** Charlotte Gaskell, Kim Linton, Mark Bishton, Graham McIlroy, Siân Lax, Sonia Fox, Louise Hopkins, Rebecca Collings, Malcolm Rhodes, Tania Seale, Aimee Jackson

**Affiliations:** 1 Cancer Research UK Clinical Trials Unit, University of Birmingham, Edgbaston, Birmingham, B15 2TT UK; 2 Department of Medical Oncology, The Christie NHS Foundation Trust, Manchester, M20 4BX UK; 3 Division of Cancer Sciences, The Manchester Cancer Research Centre, University of Manchester, Manchester, M20 4GJ UK; 4 Translational Medical Sciences, University of Nottingham, Nottingham, NG7 2RD UK; 5 Department of Haematology, Nottingham University Hospitals NHS Trust, Nottingham, UK; 6 Patient advocate, National Cancer Research Institute, London, UK; 7 Division of Cancer Sciences, Wolfson Molecular Imaging Centre, The University of Manchester, 27 Palantine Road, Withington, Manchester, M20 3LJ UK

**Keywords:** Bayesian, Power priors, Trial design, Novel therapies, Follicular lymphoma

## Abstract

**Background:**

REFRACT is a randomised trial aimed at rapidly evaluating multiple novel therapies against standard treatment for relapsed or refractory follicular lymphoma (rrFL) using a minimal number of patients. To this end, we designed a prospective, adaptive, sequentially randomised clinical trial to allow multiple novel therapies to be assessed sequentially against a control arm of investigator choice standard therapy (ICT).

**Methods:**

REFRACT uses a Bayesian power priors approach enabling the sharing of control arm data from previous treatment rounds. The design allows for the randomisation ratio to be changed and fixed to 1:4 in later treatment rounds resulting in fewer patients being recruited to the control arm.

**Results:**

Following extensive simulations, we arrived at the selected design of three sequential treatment rounds, each with a control group and a novel experimental arm assessed for the primary outcome of complete metabolic response (CMR) at 24 weeks. Patients in Round 1 are randomised using a 1:1 allocation, with Rounds 2 and 3 randomised using a 1:4 allocation, in favour of experimental treatment. Using Bayesian power priors, data from control patients in earlier rounds will be shared to improve the operating characteristics in the current round. Previous control arm patients will be weighted at 75% of an active control patient within the prior, with opportunities for adjustment should control treatments change over time.

**Conclusions:**

With the use of power priors and an adaptive design this trial will sequentially evaluate three novel treatment regimens in a disease that urgently requires additional treatment options. REFRACT opened to recruitment in July 2023.

**Trial registration:**

EudraCT: 2022–000677-75; 10-Feb-2022.

ClinicalTrials.gov: NCT05848765; 08-May-2023.

## Background

Follicular lymphoma (FL) is a common subtype of low-grade non-Hodgkin lymphoma. Despite significant improvements in first-line treatment, most patients with advanced stage disease continue to experience multiple relapses and remain incurable [1]. There is a high need to develop novel therapies for relapsed or refractory FL (rrFL), as many patients acquire resistance to standard chemotherapy over time or experience early treatment failure leading to inferior survival [2]. There are currently no standard treatment pathways and few randomised trials to guide therapy. Many promising drugs are in clinical development for rrFL, however most trials are either single arm designs or randomised against a non-standard comparator arm [3]. The relative efficacy and safety of novel agents, and their role and sequencing in modern treatment pathways, is poorly understood. The REFRACT trial is designed to address this gap.

Whereas a traditional frequentist framework used in the design and analysis of randomised controlled trials would provide evidence of statistical significance using p-values centring on the hypothesis that there is no treatment effect present, Bayesian statistics offer an alternative framework, allowing for the incorporation of prior knowledge (priors) of outcomes. This can increase the accuracy of probabilities reported for each outcome and reduce the size of the required study compared with frequentist approaches [4–6].

While designs, such as the MASTER design [7], present methods to assess multiple therapies in a single arm sequential framework and improve efficiency, extending this concept to a randomised questions allows potential for further benefits in efficacy by applying the power priors design [8].

As described within this paper, we designed an efficient randomised trial that allows for rapid sequential evaluation of multiple emerging novel therapies[9]. REFRACT utilises a Bayesian approach to share control data whilst simultaneously reducing the number of patients needed to be recruited—an important factor to support feasibility within a relatively rare disease.

## Methods

### The REFRACT trial

The primary aim of REFRACT is to identify novel therapies for patients with rrFL with superior efficacy compared to standard immuno-chemotherapy based on post-treatment induction complete metabolic response (CMR) rate assessed by PET-CT at 24 weeks using the Deauville 5-point scale and Lugano 2014 criteria [10]. Key secondary outcomes include overall metabolic response (CMR + partial metabolic response (PMR) by PET-CT at 24 weeks); progression free survival (PFS) defined as the time from randomisation to the date of first disease progression or death from any cause; overall survival (OS) defined as time from randomisation to the date of death from any cause; and duration of complete response (DoCR) defined as the time from complete metabolic response by PET-CT to relapse/progression or death from any cause. For details see [9].

### Bayesian design

REFRACT is a prospective phase II Bayesian platform study with three sequential treatment rounds utilising power priors to share data from the control arm of earlier treatment round(s) with later rounds. Each treatment round will evaluate the comparison of experimental treatments versus investigator choice therapy (ICT) for rrFL; for details see [9]. Patients in Round (R)1 will be randomised using a 1:1 allocation ratio to receive either ICT or experimental treatment with epcoritamab + lenalidomide. Patients in R2 and R3 (experimental treatments yet to be selected) will be randomised using a 1:4 allocation ratio in favour of the experimental treatment [9].

In the evaluation of the primary outcome for this study, the PET-CMR rate at 24 weeks will be compared between the control arm and each sequential experimental treatment. We consider an improvement in PET-CMR rate of 15% in the experimental arm compared with control to be clinically meaningful. The experimental arm will be deemed to be successful and warranting further investigation if there is a 60% or greater probability that the true difference between treatment arms is 15% or greater. 

$$Prob\left(true\;difference\;between\;arms\geq15\%\right)\geq60\%$$

In this phase II setting, we are looking for evidence that novel therapies require further investigation, as such we chose a lower than usual probability to determine whether a treatment requires further investigation or not. This decision was made due to the current lack of research and data available to determine a standard of care, knowledge that the current available treatments have low response rates within this patient population, in addition to current lack of available novel treatment options being researched within this rare disease. As such, in this situation we are willing to accept a higher chance of taking an ineffective treatment forward for further investigation compared to a higher chance of rejecting a potentially effective treatment.

A total sample size for all three treatment rounds of 284 patients (95 control + 189 experimental arm patients in total) was chosen based on feasibility assessments. R1 will treat 126 patients (63 patients per arm). R2 and R3 will each treat 16 patients in the control arm and 63 in the experimental arm. To make the most efficient use of available patients and reduce the number of control arm patients required in R2 and R3, data from patients recruited to previous control arms will be incorporated into subsequent rounds using power priors, increasing the effective sample size of R2 and R3 [8]. The recruitment of patients to the control arm is maintained through R2 and R3 to eliminate the biases associated with the use of historical controls, single-arm, and non-randomised trial designs. Additionally, this methodology allows for weighting of this incorporated information, dependent upon the similarity of the control groups, as described in the “Weighting of control data” subsection.

This design, as well as allowing for weighting of control data in subsequent analyses, allows for efficient evaluation of the results following completion of each round. For each round, once recruitment has completed and all patients have met the primary outcome definition the final analysis will be conducted and reported upon. Meaning, for example, as recruitment is ongoing in R2, the efficacy of the novel therapy in R1 will be assessed and published. For each round, we have two pre-planned analyses, the first being the analysis of the primary outcome measure and the second the analysis of outcomes with longer term follow-ups such as PFS and OS.

### Sample size

Due to the use of Bayesian methodology, statistical alpha and power were not calculated, instead sample size justifications were performed by calculating the probability that the true PET-CMR rate in the experimental arm is greater than that in the control arm. Operating characteristics were produced through simulation, to assess the probability of drawing the correct conclusions, under predefined conditions. The simulations conducted cover four main scenarios; Scenario 1: a higher response rate is observed in the control group compared with the experimental arm; Scenario 2: no difference between the response rate in the control and experimental group is observed; Scenario 3: exactly a 15% difference in response rates (in favour of the experimental arm; with a difference of 15% thought to be clinically meaningful) is observed; and Scenario 4: A larger than 15% difference in response rates (in favour of the experimental arm) is observed. Simulations for all three rounds were conducted for control rates ranging between 40 and 60%, certainty levels ranging from 50 to 80%, looking for a ≥ 15% difference in response rates, with operating characteristics produced from 10,000 simulations. For R2 and 3, simulations were conducted for each permutation of the preceding randomisation(s) estimated control rate and the estimated control rate in the current randomisation, applying weightings to previous data of 0.75 and 0.5. There are no formal statistical interim analyses planned for the REFRACT trial.

### Weighting of control data

The power priors design allows us to ‘weight’ borrowed control data, with a weighting of 1 indicating full borrowing (i.e., every patient is worth 1 patient in the subsequent analysis) and a weighting of 0 indicating no borrowing.

A priori in the REFRACT study, the weighting is predefined at 0.75, meaning each control patient is worth 75% of a patient in the subsequent randomisation(s). This weighting was chosen to reflect the clinical opinion that the standard treatments and patient population during the lifetime of the trial would remain largely unchanged (or kept unchanged within the eligibility criteria for the trial); full borrowing i.e., setting the weight at 1, was felt to be inappropriate within this trial due to the time difference in recruitment to each round. It was felt, both clinically and statistically, that within this patient population the use of 0.75 as the pre-defined weighting allowed for some penalisation of the potential bias in using “historical control data” whilst still accounting for the clinical similarities that are expected due to the lack of treatment advancement in this disease area.

Throughout R2 and R3 every data monitoring committee (DMC) report will include baseline and response information on both the control arm data that will be included and the current recruiting round patients reported side by side in order to allow the DMC to check that the patient populations remain similar and if there is any drift in response rates being observed. If there is a scenario in which substantial drift in the patient population or standard of care treatment between rounds is observed, the independent DMC will have the opportunity to review the weightings and suggest adjustments. Prior to any analysis of R2 and R3, a weighting decision report will be shared with the DMC; this report will clearly detail the choice of weighting deemed to be appropriate for the analysis including clear justification as to why this weighting was chosen. This choice will be determined through comparison of baseline characteristics (no planned hypothesis testing), details of any changes to standard of care, and time between closure of the previous rounds’ recruitment and closure of the current rounds’ recruitment. This recommendation will then be fed back to the trial steering committee (TSC) and a final decision on weightings made. Irrespective of the weight used within the analysis, extensive predefined sensitivity analyses will be conducted to assess the suitability of the weight used within the main analyses, this will be reanalysis with lower weights, should a weighting of 0.75 be implemented, or reanalysis with higher weightings should a weighting lower than 0.75 be implemented. Extensive simulations have been carried out under various scenarios and weightings to assess the impact a change in weightings will have on the trials operating characteristics.

## Results

**Fig. 1:**
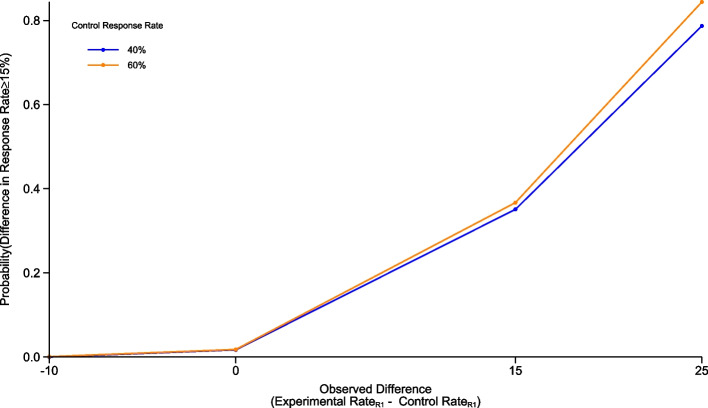
Round 1 operating characteristics. Graph showing the simulated operating characteristics for Round 1. The operating characteristics presented are for the estimated control response rates of 40% and 60%, calculated for estimated response rate differences of −10% (control group is superior), 0%, 15% and 25% compared to the estimated experimental response rate.

For R1 we employed a conjugate Beta-Binomial analysis with a non-informative prior of Beta(1, 1). The probabilities calculated indicate the certainty that the true difference in PET-CMR rate is ≥ 15% based on our simulated CMR rates. Calculations were performed to detect this difference compared with control group PET-CMR rates of 40, 50, and 60%; the obtained operating characteristics are presented for an estimated 40% and an estimated 60% control group PET-CMR rate against the estimated PET-CMR rate in the experimental group across the four scenarios described within the methods (Fig. 1).

In the scenario where we look to observe a true difference between treatment arms of ≥ 15% based on a 40% PET-CMR rate in the control group and 126 patients recruited (i.e., 26/63 responses): if a difference of 25% in PET-CMR between treatment arms is observed there is a 79% probability that the true difference in response rates is ≥ 15%. In scenarios with a 60% PET-CMR rate in the control group, the probability that the true difference is ≥ 15% is 84% when a 25% difference is estimated.

Similarly, for R2 and 3 we employed the same conjugate Beta-Binomial analysis, however in these simulations the prior was informed using control rates from previous rounds. These simulations used the same parameters used for R1 and, a range of borrowing between 0.5 and 0.75.

**Fig. 2:**
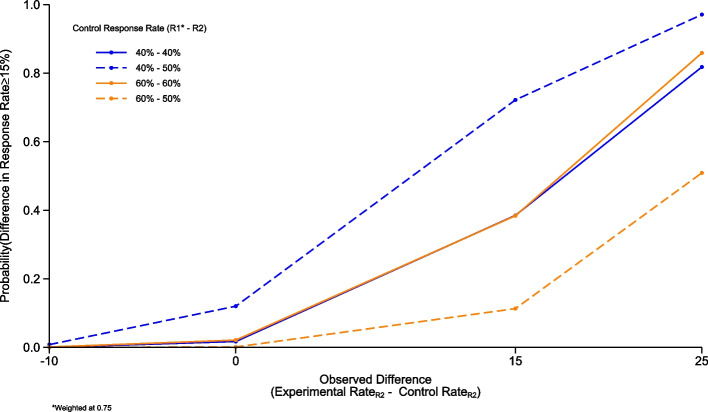
Round 2 operating characteristics. Graph showing the simulated operating characteristics for Round 2. The operating characteristics presented are for the estimated control response rates of 40% and 60% within R1 (weighted at 0.75) and 40%, 50%, and 60% within R2, calculated for estimated response rate differences of −10% (control group is superior), 0%, 15% and 25% compared to the estimated experimental response rate.

In the scenario where we look to observe a true difference between treatment arms of ≥ 15% based on a 40% PET-CMR rate in the R1 control group (26/63 responses), 40% PET-CMR rate in the R2 control group (7/16 responses) and 79 patients recruited, if a difference of 25% in PET-CMR between treatment arms in R2 is observed and we weight R1 control data at 0.75 there is an 82% probability that the true difference in response rates is ≥ 15% (Fig. 2). This drops to 80% probability under the same scenario when weighting control patients from R1 at 0.5.

**Fig. 3:**
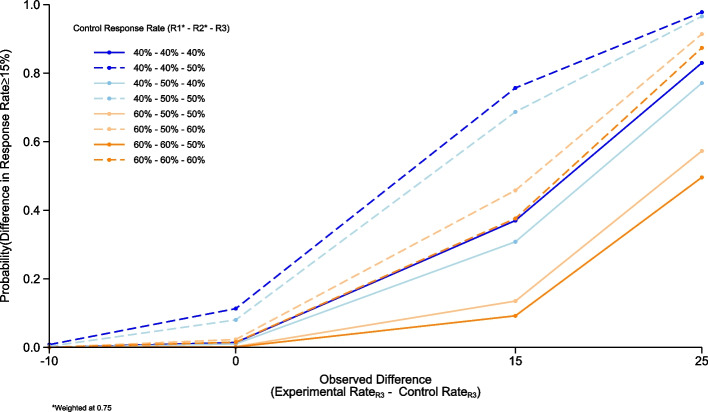
Round 3 operating characteristics. Graph showing the simulated operating characteristics for Round 3. The operating characteristics presented are for the estimated control response rates of 40% and 60% within R1 (weighted at 0.75); 40%, 50%, and 60% within R2 (weighted at 0.75) and 40%, 50%, and 60% within R3, calculated for estimated response rate differences of −10% (control group is superior), 0%, 15% and 25% compared to the estimated experimental response rate.

**Fig. 4:**
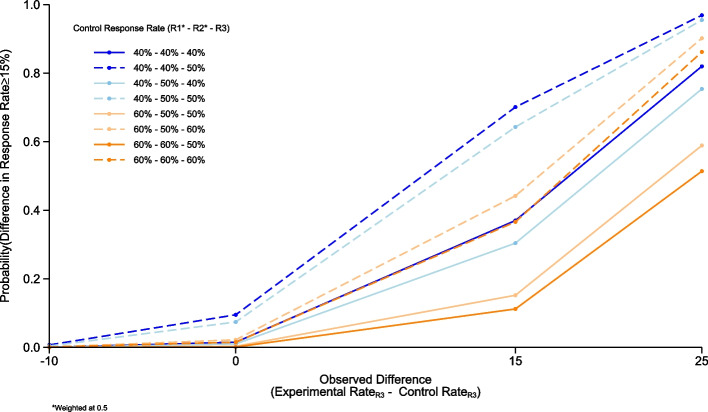
Round 3 operating characteristics. Graph showing the simulated operating characteristics for Round 3. The operating characteristics presented are for the estimated control response rates of 40% and 60% within R1 (weighted at 0.5); 40%, 50%, and 60% within R2 (weighted at 0.5) and 40%, 50%, and 60% within R3, calculated for estimated response rate differences of −10% (control group is superior), 0%, 15% and 25% compared to the estimated experimental response rate.

The simulations for R3 showed that in the scenario where we observe a 40% control rate in both R1 and R2, borrowing at a weighting of 0.75, a 40% control rate in R3 and a difference of 25% in PET-CMR between the R3 control and experimental patients, gives an 83% probability that the true difference in response rates is ≥ 15% (Fig. 3). This drops to 82% probability if we weight previous control patients at 0.5. (Fig. 4) Simulations for this design were all conducted in R version 3.6.0.

## Discussion

The power priors design implemented in the REFRACT trial facilitates the sequential assessment of novel treatments in a disease setting where patient numbers are low and multiple novel therapies are available.

Several different possible designs were considered for this study to account for the rare nature of the disease and the desire to conduct a randomised trial evaluating multiple treatment arms. The aims of the power priors design are like those of the multi-arm multi-stage (MAMS) designs and therefore this alternative design was considered during the early design process of REFRACT. Implementation of a MAMS design would have advantages such as allowing for multiple experimental arms to be opened to recruitment simultaneously, resulting in a similarly low sample size (N = 252 if all four arms were opened at the same time). However, in this disease where there are insufficient patients to simultaneously evaluate multiple therapies without extending recruitment to a multi-national setting, it is more feasible to add additional experimental arms in a sequential way. Furthermore, treatment options currently under consideration for R2 and R3 are in early phase clinical development, pending recommended doses for phase II evaluation. Implementation of a MAMS design under these conditions would have required a far larger number of concurrent control patients and therefore an increase in sample size beyond that deemed feasible.

Furthermore, given the rarity of rrFL in the UK, it would take as long to recruit to three experimental arms concurrently as it would sequentially. In this context, the sequential design has the benefit of allowing primary outcome data to be published for each experimental-control comparison as they mature, instead of at the end of the trial as would be required with concurrent experimental arms. This rapid analysis and communication of results will accelerate the identification of promising treatment regimens—a core ambition of REFRACT shared by patient groups, investigators and industry partners.

Additionally, we considered various options for the control arm for this study. Historical controls were initially considered for the control arm comparison, due to the lack of advancement in treatments and similar patient populations. However, there is a lack of appropriate data available in this patient population for the current treatment options to make this a viable option as a comparator arm. One aim from the control arm within the REFRACT trial, is to evaluate clinical outcomes for ICT to provide a benchmark and data for these treatments. We considered using a standard 1:1 randomisation across all three arms, this would allow for direct comparisons without borrowing of previous control information, however this would increase the sample size by 94 patients, which in this population would’ve increased the overall recruitment time by at least 12 months which, as this trial aims to rapidly evaluate treatments for this patient population was not desirable. A 4:1 randomisation was chosen as we wanted to retain the experimental arm sample size at 63 patients, as per R1, whilst reducing the recruitment time overall for the study. The 4:1 randomisation ratio allows for quicker completion of recruitment in R2 & R3 (30 months in R1, versus 17 months in R2 and R3), as well as increasing the probability within this high-risk disease of obtaining access to novel therapies whilst still retaining a randomised element to the trial and analysis. Alternative randomisation allocations would’ve increased the trial duration and time to treatment evaluation. Additionally, although the control sample size for the later rounds are 16 patients, the effective sample size, under the current proposed 0.75 weightings will be 63 patients which is in alignment with the R1 sample sizes.

There are two possible power prior designs that can be used, these are fixed or dynamic power priors, in the REFRACT trial, we opted to implement fixed power priors. A fixed power prior, uses a pre-defined parameter to determine the level of borrowing that will be used when constructing the prior distribution, this is different to the dynamic power prior in which the level of borrowing is adapted based on the similarity between the current data and that being borrowed from, there are a number of ways this adaptation is managed, one of which is the use of Hellinger distance, which has been shown to have advantages over other borrowing methods in non-inferiority trials [11]. The decision to used fixed power priors in this trial was taken due to the anticipated homogeneity of our control patients across rounds, as such it was felt that the added complexity of determining dynamic power priors was not required within this design and patient population.

Whilst minimising the sample size, the lack of a full set of concurrent control patients to use in comparisons for treatment R2 and 3 is also a limitation of the REFRACT design, especially as changes to the patient population or current standard of care during the study will increase the risk of bias. Historically, and indeed currently, treatment of rrFL is an area of unmet need, and very few novel treatments were approved in the last decade. Given the regulatory framework of drug approvals in the UK, and after horizon scanning of agents under development, new treatments are likely to be approved but a substantial change in care for rrFL patients is not anticipated during the lifetime of this trial – a view shared by UK investigators and independent peer reviewers of the trial design. One advantage of using the power priors design, is the ability to weight borrowed control data, this allows for increased flexibility during the trial to account for unanticipated changes within the patient population and/or disease pathway reducing the introduction of potential biases of using “historical” control data.

We nevertheless considered a scenario where new approvals lead to substantial changes to standard treatment. We considered updating the list of permitted therapies within the control arm for later rounds but decided against this in scenarios where the new treatment was significant more effective (e.g. CAR-T therapy) compared to those in previous rounds as this could lead to unreliable control rates in the analysis. This could be counteracted by reducing the weightings; however, it was felt that the reduction in weighting would likely have to be close to 0 (no borrowing) in such a scenario to avoid introducing bias; as such, updating the allowed standard of care treatments during the trial was not a feasible option. Where standard of care has changed and treatment effectiveness is similar or slightly higher than currently planned, changes to the control arm may be permitted upon careful consideration and approval from the DMC and TSC.

The REFRACT trial was co-developed, from inception, with the EMERGE patient and public involvement (PPI) group [9]. The clear consensus of the group was to prioritise the accelerated development of novel treatments. They reviewed and refined the design; specifically, their feedback supported the use of a novel design to maximise efficiency and reach trial read-outs quickly. PPI representatives are members of the Trial Steering Committee responsible for supervising the conduct of the trial and monitoring its progress.

## Conclusions

Using a primary outcome of response assessment at 24 weeks, and methods such as power priors and an adaptive design to minimise patient numbers, the REFRACT trial will sequentially evaluate three novel treatment regimens in the shortest timeframe possible for a disease that urgently requires new treatment options.

## Data Availability

Statistics code is available to be shared, requests should be made to the trial management group via the corresponding author.

## Abbreviations

CMR: Complete metabolic response
DMC: Data monitoring committee
DoCR: Duration of complete response
FL: Follicular lymphoma
ICT: Investigator choice standard therapy
MAMS: Multi-arm, multi-stage
OS: Overall survival
PET-CT: Positron emission tomography-computed tomography
PFS: Progression-free survival
PMR: Partial metabolic response
PPI: Patient and Public Involvement
R: Round
rrFL: rRelapsed/refractory FL
TSC: Trial steering committee
